# A First Phylogeny of the Genus *Dimocarpus* and Suggestions for Revision of Some Taxa Based on Molecular and Morphological Evidence

**DOI:** 10.1038/s41598-017-07045-7

**Published:** 2017-07-27

**Authors:** Suparat K. Lithanatudom, Tanawat Chaowasku, Nattawadee Nantarat, Theeranuch Jaroenkit, Duncan R. Smith, Pathrapol Lithanatudom

**Affiliations:** 1 Program in Genetics, Faculty of Science, Maejo University, Chiang Mai, 50290 Thailand; 2 Center of Excellence in Bioresources for Agriculture, Industry and Medicine, Department of Biology, Faculty of Science, Chiang Mai University, Chiang Mai, 50200 Thailand; 3 Department of Horticulture, Faculty of Agricultural Production, Maejo University, Chiang Mai, 50290 Thailand; 4 Institute of Molecular Biosciences, Mahidol University, 73170 Nakorn Pathom, Thailand

## Abstract

*Dimocarpus longan*, commonly known as the longan, belongs to the family *Sapindaceae*, and is one of the most economically important fruits commonly cultivated in several regions in Asia. There are various cultivars of longan throughout the Thai-Malay peninsula region, but until now no phylogenetic analysis has been undertaken to determine the genetic relatedness of these cultivars. To address this issue, 6 loci, namely ITS2, *matK*, *rbcL*, *trnH-psbA*, *trnL-I* and *trnL-trnF* were amplified and sequenced from 40 individuals consisting of 26 longan cultivars 2 types of lychee and 8 herbarium samples. The sequencing results were used to construct a phylogenetic tree using the neighbor-joining (NJ), maximum likelihood (ML) and Bayesian inference (BI) criteria. The tree showed cryptic groups of *D*. *longan* from the Thailand-Malaysia region (*Dimocarpus longan* spp.). This is the first report of the genetic relationship of *Dimocarpus* based on multi-locus molecular markers and morphological characteristics. Multiple sequence alignments, phylogenetic trees and species delimitation support that *Dimocarpus longan* spp. *longan* var. *obtusus* and *Dimocarpus longan* spp. *malesianus* var. *malesianus* should be placed into a higher order and are two additional species in the genus *Dimocarpus*. Therefore these two species require nomenclatural changes as *Dimocarpus malesianus* and *Dimocarpus obtusus*, respectively.

## Introduction

*Dimocarpus* is a genus belonging to the family *Sapindaceae*, also known as the soapberry family of flowering plants (Angiospermae)^1^. The major characteristics of this genus are trees or shrubs which can grow up to 25–40 meters (m) tall with pinnate leaves. The flowers are seen as large panicles. The edible fruit is 3–5 centimeters (cm) long containing a single seed surrounded by a layer of fruit pulp^2^. *Dimocarpus* is primarily distributed in tropical South and Southeast Asia, ranging from Sri Lanka and India to East Malaysia and Australia^3–6^. The well-recognized edible fruits derived from this genus known as longan are produced from *Dimocarpus longan*.

The most recent revision of the genus *Dimocarpus* was published in 1971, with additional minor modification in 1994^2, 5^. According to Leenhouts (1971 and 1994), this genus comprises of only 6 species, namely *Dimocarpus australianus* (1973)^6^, *Dimocarpus dentatus*, *Dimocarpus foveolatus*, *Dimocarpus fumatus*, *Dimocarpus gardneri* and *Dimocarpus longan*. Furthermore, from 1974 to 1983, an additional 3 species have been included, namely *Dimocarpus yunnanensis* (1977)^7^, *Dimocarpus confinis* (1979)^8^ and *Dimocarpus leichhardtii* (1983)^9^ giving a current total of 9 species. However, the latest three proposed new species remain unresolved and therefore further research is needed to draw definitive conclusions about this genus. In addition, there are 6 subspecies (spp.) identified as part of this genus. Four of them belong to *Dimocarpus fumatus* while the other 2 subspecies are from *Dimocarpus longan* ^5, 6, 10^. Of these, only *Dimocarpus longan* has its own variety. Two varieties including var. *malesianus* and var. *echinatus* belong to spp. *malesianus*, the other three varieties, var. *obtusus*, var. *longan* and var. *longepetiolulatus*, are all members of the spp. *longan* ^10^.

Commonly known as longan, *Dimocarpus longan* is the most well-known and important species from this genus. It produces an edible fruit and is widely cultivated in tropical and sub-tropical Asian countries such as China, Taiwan, Vietnam and Thailand^11^. In general, longan products are exported as fresh fruit or are processed to dried fruit which can be further processed to longan juice or syrup^11^. Nowadays, the demand for longan is rising due not only to the recent discovery of proposed medicinal properties of this fruit such as enhancing memory, promoting blood metabolism, relieving insomnia and preventing amnesia, but also because of the proposed beneficial activities of secondary metabolites from longan such as anti-oxidative, anti-obesity, anti-cancer, anti-tyrosinase, and immune-modulatory activities^12–15^.

In China alone, more than 400 cultivars of *Dimocarpus longan* have been reported^16^. In contrast in Thailand, one of the world’s largest exporters of longan^17^, 26 cultivars are commonly grown for domestic consumption and export. In particular, there are 25 cultivars of *Dimocarpus longan* spp. *longan* var. *longan* and one cultivar characterized as *Dimocarpus longan* spp. *longan* var. *obtusus* (commonly referred to as “Thao” by Thai people). The most commonly planted cultivars in Thailand are E-Daw, Chompoo, BiewKhiew Chiangmai, Haew, Baidom and Phetsakorn. Each cultivar is named according to its origin and morphological characters and/or the name of breeder or discoverer^18^.

Given the agricultural and medicinal significance of longan, a number of studies have tried to develop molecular markers to assess the diversity of the numerous longan cultivars grown locally in China, Indonesia and Thailand as well as in germplasm collections from various regions. Such markers could potentially assist breeding program and facilitate authentication strategies such as, Random Amplified Polymorphic DNA (RAPD)^19^, Amplification Fragment Length Polymorphism (AFLP)^20^, Inter-Simple Sequence Repeat (ISSR)^21^ and Single Nucleotide Polymorphism (SNP)^17^. Surprisingly however, no study has determined the genetic relationships between longan cultivars coupled with an evolutionary (phylogeny) analysis, or assessed the results in relationship to other taxa of the genus *Dimocarpus*. Furthermore, it should be noted that even the latest revision to genus *Dimocarpus* (1994) was based solely on morphological data^2^. The acquisition of molecular data is therefore necessary to prove and/or support the previous taxonomic classification of this particular genus.

In this study, we aimed to investigate the evolutionary relationship of the genus *Dimocarpus* including longan cultivars (*Dimocarpuslongan spp*. *longan*) commonly grown in Thailand and determine the validity of species boundaries in *Dimocarpus* by combining multi-gene molecular phylogeny and morphological approaches. In addition, we use species delimitation methods to gain insights into species designations of the possibly confounding morphological characters used for *Dimocarpus* taxonomy. These results should be of high interest to academics concerned about the future genetic conservation of *Dimocarpus* in the Thai-Malay peninsula region.

## Results

### Data analysis

**Figure 1:**
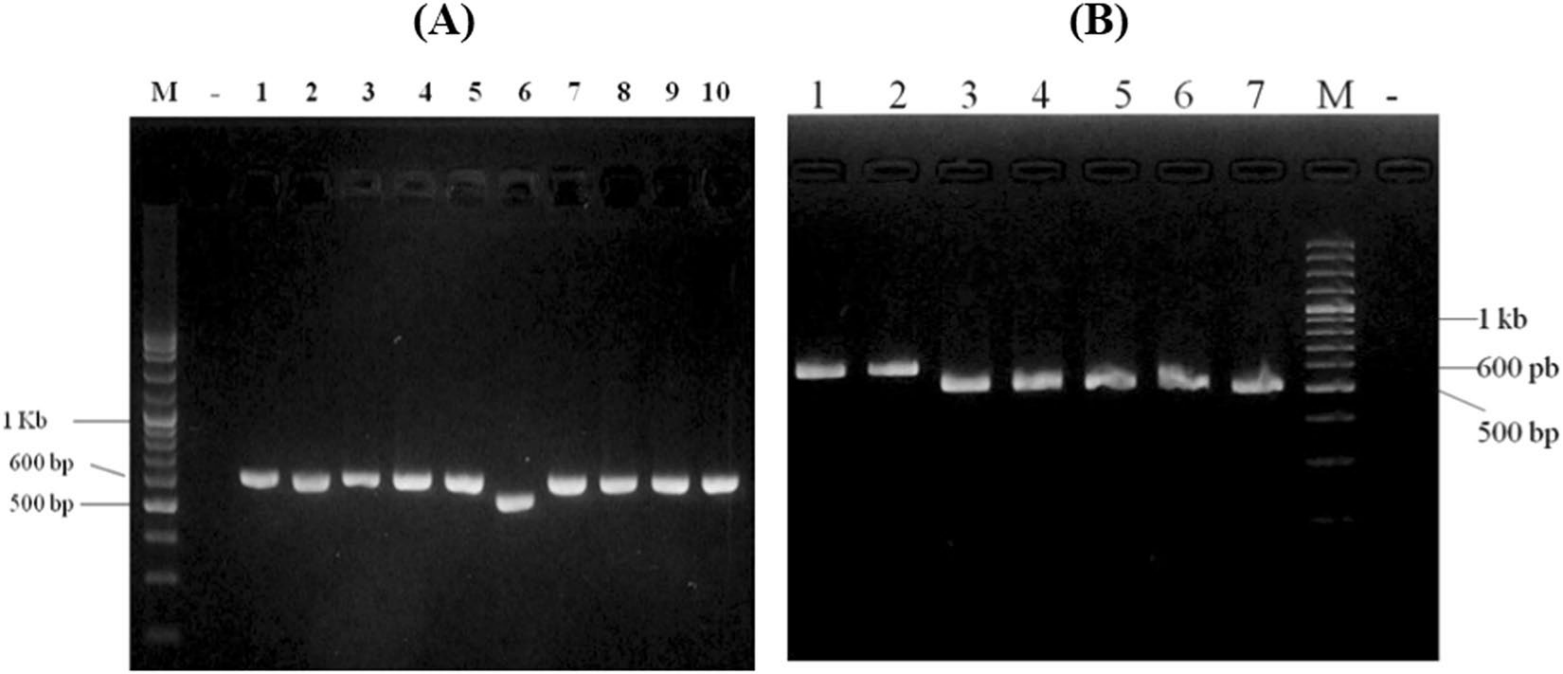
(**A**) The PCR fragment amplified using trnH-psbA primers. The smaller *trnH-psbA* PCR fragment was identified only in the Thao sample (lane no. 6) (M = 100 bp DNA marker, − = Negative control, 1–10 = longan samples). (**B**) The short *trnH-psbA* gene fragment amplified from 5 Thao longan cultivars compared with other longan samples. The deletion of *trnH-psbA* gene was detected in all of the Thao samples (lane 3–7) as compared with other longan cultivars (M = 100 bp DNA marker, − = Negative control, 1 = E-Daw, 2 = Lychee samples, 3–7 = Thao).

The sizes of PCR products amplified from ITS2, matK, rbcL, trnH-psbA, trnL-I and trnL-trnF primer were about 300, 690, 540, 520, 340 and 380 base pairs (bp), respectively. Observation of PCR products after electrophoresis though 1.5% agarose gels revealed different product sizes of the *trnH-psbA* PCR fragment amongst the longan samples (Fig. 1A). The different PCR product sizes may be due to an InDel mutation which was found only in *Dimocarpus longan* spp. *longan* var. *obtusus* (Thao) (lane no. 6 in Fig. 1A). The PCR amplification was performed with another 4 DNA samples extracted from different longan trees which were all Thao cultivar, and the results showed a smaller *trnH-psbA* PCR fragment in all samples in comparison with other longan cultivars (Fig. 1B).

**Figure 2:**
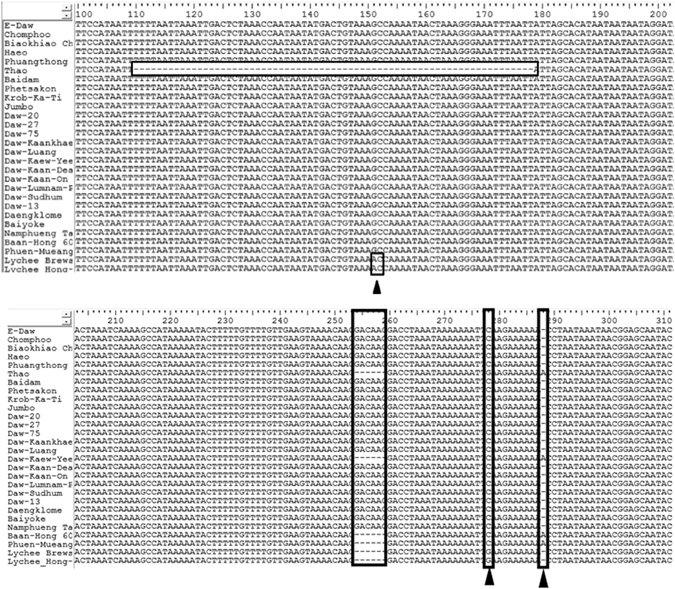
Multiple sequence alignment result of *trnH-psbA* gene. The InDel mutation is in the box and base substitution are indicated by black triangles.

Conservation of the *matK* and *rbcL* gene sequences was observed after multiple sequence alignment. However, significant diversity was observed in the *trnH-psbA* gene amongst the 26 longan and 2 lychee cultivars. Three locations of InDel mutations and 2 locations of nucleotide substitution were found after the multiple sequence alignment. Interestingly, a 70 nucleotide deletion at position 109 to 178 was observed only in the Thao cultivar. A six nucleotide deletion at position 254 to 259 was found in Thao, Daw Kaew Yee, Baan-Hong 60, Phuen-Mueang and the 2 lychee cultivars. An adenine base insertion at position 289 was found in 4 longan cultivars, namely Thao, Daw Kaew Yee, Baan-Hong 60 and Phuen-Mueang. An adenine base substitution was detected in only 2 lychee cultivars whereas a guanine base substitution was found in the 2 lychee samples and the Thao cultivar. Moreover, a six base pair deletion was found in Thao, Daw Kaew Yee, Baan-Hong 60 and Phuen-Mueang samples. A second nucleotide substitution (guanine; G) occurred at position 277 in Thao and the 2 lychee samples. Finally the insertion of an adenine was detected in the Thao, Daw Kaew Yee, Baan-Hong 60 and Phuen-Mueang cultivars. The result of multiple sequence alignment of *trnH-psbA* gene is shown in Fig. 2.

### Phylogenetic analysis

A total of 40 individual samples (including out groups) consisting of 3 species 2 subspecies and 26 longan cultivars were used to reconstruct the phylogenetic trees based on the nuclear ITS2 region and 5 plastid markers (*matK*, *rbcL*, *trnH-psbA*, *trnL-i* and *trnL-trnF*). A partition homogeneity test by PAUP 4.0b10, using 100 replicates^22^ showed no significant differences were found between markers (P = 0.095). The uncorrected p-distance between the taxa ranged from 0.003 to 0.023 [inter/intraspecific p-distances = 0.013 and 0.001, respectively].

**Figure 3:**
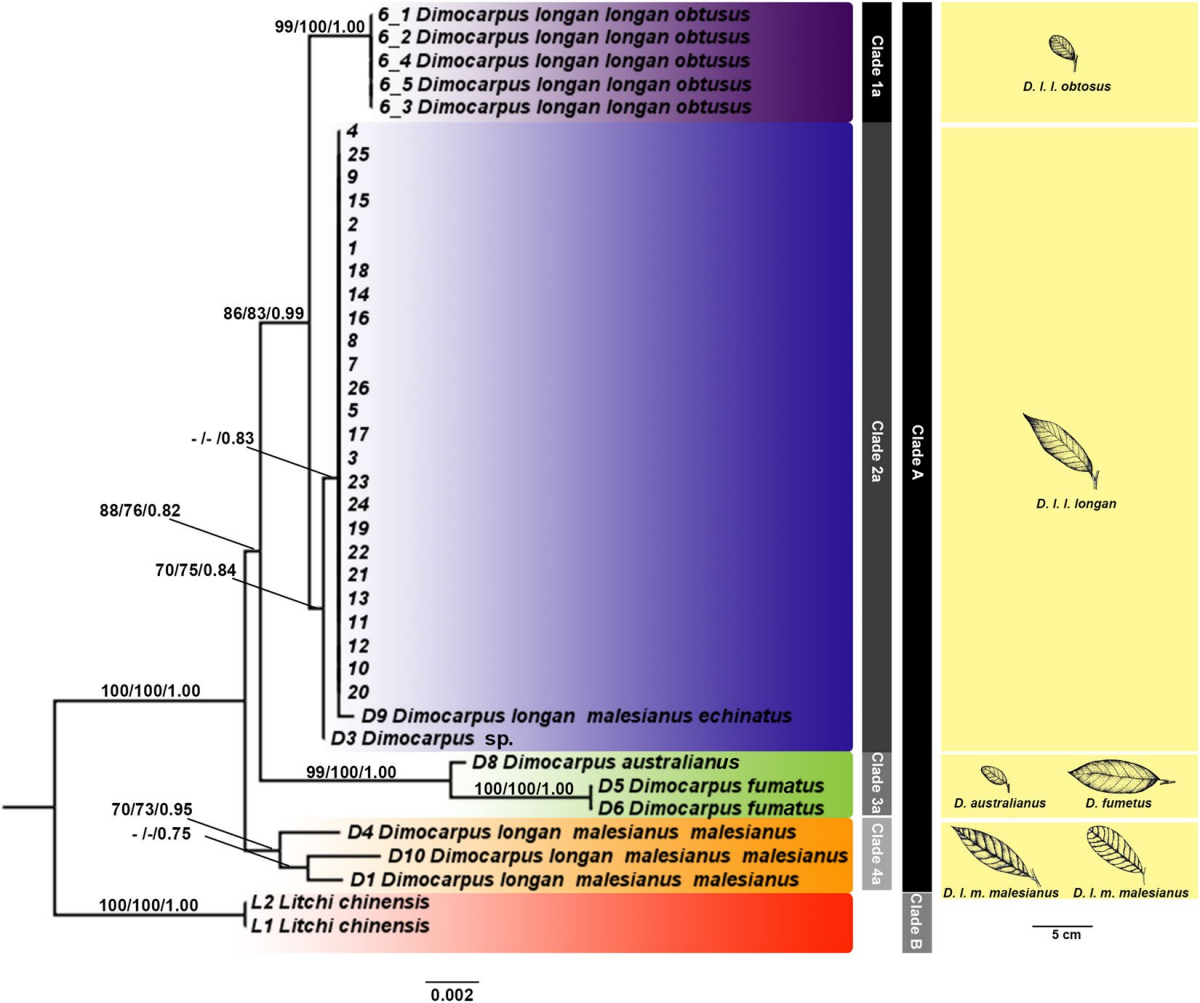
Combined gene phylogenetic tree for *Dimocarpus*. Combined gene (ITS2, *matK*, *rbcL*, *trnH-psbA*, *trnL-i* and *trnL-trnF*) Maximum likelihood tree for *Dimocarpus*. We provide neighbor-joining, maximum likelihood (bootstrap support, B) and Bayesian Inference (posterior probability, PP) support values for each node, respectively.

The phylogeny based concatenate of nuDNA and chDNA showed the evolutionary relationship among *Dimocarpus* and its position (Fig. 3). All trees from each DNA dataset were almost congruent in topology. The phylogenetic tree was divided into two main clades with high statistical support (Clade A and Clade B in Fig. 3) with 100, 100 of NJ and ML bootstraps and 1.00 of BI support. *Dimocarpus* was monophyletic and well separated from the out group. Clade A also divided into 4 sub-clades as Clade 1a, 2a, 3a and 4a with moderate to high statistical support (Fig. 3). The 4 sub-clades consist of *D*. *longan* spp. *longan* var. *obtusus* (Thao) (Clade 1a in Fig. 3) with 99, 100 of NJ and ML bootstraps and 1.00 of BI support, the *D*. *longan* spp. *longan* var. *longan* were grouped together (Clade 2a in Fig. 3) with 0.83 of BI support, *D*. *autralianus* (from Australia) and *D*. *fumatus* (from Malaysis) were grouped together (Clade 3a in Fig. 3) with 99, 100 of NJ and ML bootstraps and 1.00 of BI support and *D*. *longan* spp. *malesianus* var. *malesianus* (Clade 4a in Fig. 3) with 70, 73 of NJ and ML bootstraps and 0.95 of BI support. The result shows that *Dimocarpus longan* was polyphyletic and separated into three sub-clades as 1a, 2a and 4a (Fig. 3). This result shows that the taxonomy of *Dimocarpus longan* is confusing and needs to be clarified.

### Species Delimitation

**Figure 4:**
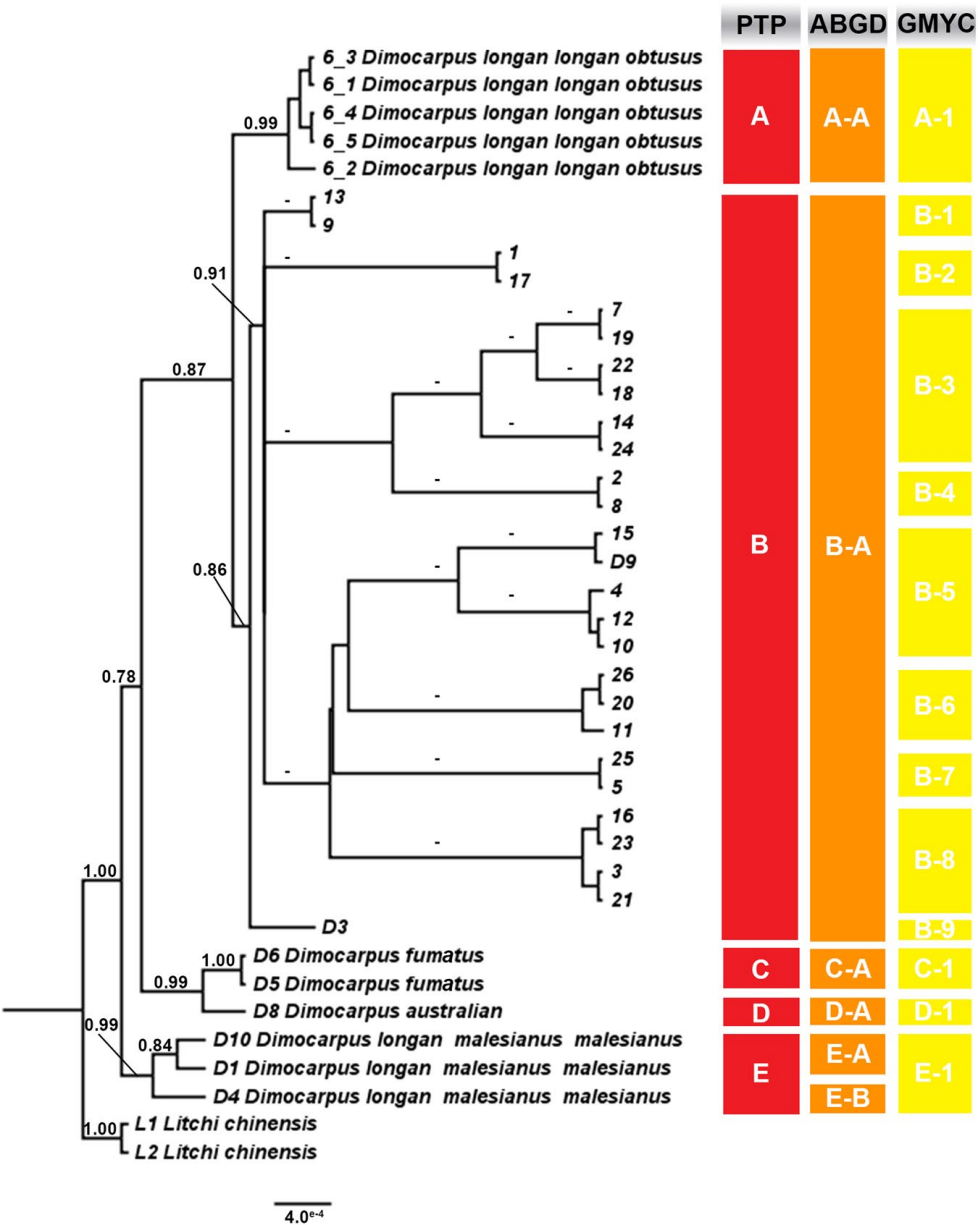
Species delimitation analyses on the concatenated dataset. The tree of species delimitation analyses was reconstructed using Poisson Tree Processes (PTP), Automatic Barcode Gap Discovery (ABGD), and General Mixed Yule Coalescent (GMYC) and was labeled with Bayesian (posterior probability, P; top) support values for each node on this Bayesian phylogenetic tree.

The PTP analysis revealed that the likelihood of the null model in that all sequences belong to a single species was found to be significantly lower than the maximum likelihood species delimitation (P < 0.001). For the PTP analysis, the results revealed delimitation of five in group PSHs, hereafter denoted PSH-A to PSH-E (Fig. 4). *Dimocarpus longan* sensulato was delimited as three PSHs (PSH-A, PSH-B and PSH-E in Fig. 4). The tree showed the cryptic groups of *Dimocarpus longan* from both Thailand and the Malaysia region. Cryptic group designations of both PTP- and ABGD-delimited PSHs labels are almost similar, except for PSH-E that ABGD-delimited divided to be 2 sub-groups (Fig. 4). The GMYC-delimited method resulted in recovery of 13 PSHs within *Dimocarpus*. The results did not conflict with PTP- and ABGD-delimited PSHs but suggested additional phylogenetic species within PTP-delimited PSHs for the *Dimocarpus* cultivars from Thailand (PSH-B in Fig. 4) and *Dimocarpus longan* spp. *malesianus* var. *malesianus* from Malaysia. The GMYC, ABGD results were almost consistent with the five PSHs identified by PTP, except for PSH-B and PSH-E.

## Discussion

The PCR product of the *trnH-psbA* gene run on 1.5% agarose gels showed a smaller PCR fragment in lane number 6 which was the Thao cultivar (*Dimocarpus longan* spp. *longan* var. *obtusus*) (Fig. 1A and B). The differentiation of the PCR product might be caused by an InDel mutation occurring inside this gene. The PCR amplification was not only performed on one sample of a Thao cultivar, but 4 more DNA samples were extracted from other Thao cultivar trees which were used to confirm the 70 bp deletion of this gene in the Thao cultivar. The *trnH-psbA* gene fragments amplified from the 5 Thao samples all showed the same size which confirmed the unique deletion of the *trnH-psbA* gene in the Thao cultivar (Fig. 1B). This unique genetic pattern which is found only in the Thao cultivar can be applied as an easy and cost effectively genetic marker to identify the Thao cultivar.

The maximum likelihood tree based on concatenation of nuDNA and chDNA (Fig. 3) revealed the relationship and positions of *Dimocarpus* spp. in Thailand and nearby countries. The examination of individual trees showed slight differences, but results were broadly consistent with the concatenated tree topologies. *Dimocarpus longan* was polyphyletic and divided into 3 sub-clades, designated as Clade 1a, 2a and 4a with moderate to high statistical support (Fig. 3). The multi-gene phylogenetic analyses, in combination with species delimitation methods, revealed evidence that *Dimocarpus longan* sensulato showed morphological cryptic diversity (Figs 3 and 4). Additionally, longan historically recognized as *Dimocarpus longan* with various subspecies and varieties (*D*. *longan spp*. *longan* var. *obtusus* (Clade 1a in Fig. 3), *D*. *longan* spp. *longan* var. *longan* (Clade 2a in Fig. 3), *D*. *longan* spp. *malesianus* var. *echinatus* (Clade 2a in Fig. 3) and *D*. *longan* spp. *malesianus* var. *malesianus* (Clade 4a in Fig. 3) segregated into different clades. This result challenges the validity of the species and subspecies of *Dimocarpus*. This cryptic diversity strongly supports the taxonomic description asa new species, albeit in concert with other characteristic such as sympatric/allopatric speciation, ecology, hybridization and morphology^23^. The phylogenetic trees were supported by three of the species delimitation approaches (PTP, ABGD, GMYC in Fig. 3) which identified additional PSHs within not only *D*. *longan* spp. *longan* var. *longan*, but also suggested that some of subspecies should be rearranged and recognized as species, especially, *D*. *longan* spp. *longan* var. *obtusus* (Clade 1a in Fig. 3 and PSH-A in Fig. 4) and *D*. *longan* spp. *malesianus* var. *malesianus* (Clade 4a in Fig. 3 and PSH-E in Fig. 4). Notably, *D*. *longan* was not monophyletic in any of our phylogenetic trees. These results are also supported by the different morphological characters between *D*. *longan* spp. *longan* var. *longan* (Clade 2a in Fig. 3 and PSH-B in Fig. 4), *D*. *longan* spp. *longan* var. *obtusus* (Clade 1a in Fig. 3 and PSH-A in Fig. 4), and *D*. *longan* spp. *malesianus* var. *malesianus* (Clade 4a in Fig. 3 and PSH-E in Fig. 4) which show large differences in morphological features such as habit, twigs, petals, fruits, petioles and rachis, and leaflets as detailed in Table 1. According to our results based on molecular and morphological approaches, it is strongly suggested that three subspecies/varieties of *the D*. *longan* species complex should be recognized as three distinct species, two of which are elevated to species rank: *D*. *malesianus* (Clade 4a in Fig. 3) and *D*. *obtusus* (clade 1a in Fig. 3), while *D*. *longan* spp. *malesianus* var. *echinatus* is reclassified as *D*. *longan* var. *echinatus* (clade 2a in Fig. 3) instead of synonymizing it with *D*. *longan* var. *longan* because of its unique long-spined fruits as well as some molecular autapomorphies, and when more DNA regions have been sequenced for all accessions, this taxon could end up outside other *D*. *longan* var. *longan*. In addition, the sample D3 which is *Dimocarpus* sp. is grouped in clade 2a together with other *D*. *longan* and it is sister to a clade of *D*. *longan* var. *longan* and *D*. *longan* var. *echinatus*; therefore, this taxon should be classified in its own variety of *D*. *longan* although the material available is not sufficient to fully support this. The longan varieties with names consisting of “Daw” such as E-Daw, Daw 20, Daw 27, Daw 75, Daw-Kaankhaeng, Daw-Luang, Daw Kaew Yee, Daw-Kaan-On, Daw-Lumnam-Ping, Daw-Sudhum and Daw 13 (Clade 2a in Fig. 3 and PSH-B in Fig. 4) might have different genetic backgrounds. This is supported by the GMYC species delimitation method, and revealed the diversity of longan cultivars in Thailand that will be useful for conservation management of this plant in the future. Finally, there is a need for more sensitive markers to be used to clarify the relationship of these longan cultivars in the future.

**Table 1:** The morphological characteristic comparison of 3 varieties of *Dimocarpus longan* ^2, 5, 6^.

Characteristic	*D. longan* spp. *longan* var. *longan*	*D. longan* spp. *longan* var. *obtusus*	*D. longan* spp. *malesianus* var. *malesianus*
Habit	tree	scandent shrub	tree
Twigs	brown to dark brown	whitish grey	brown
Petals	reduced	reduced	well-developed
Fruits	mostly pusticulate to granulate and nearly smooth, sometimes aculeate or colliculate	areolate, not granular	smooth to warty
Petioles and rachis	glabrous	glabrous	tomentose
Apex of leaflets	acute	obtuse to emarginate	acute to acuminate
Lower surface of leaflets	glabrous	tomentose	tomentose
Midrib on upper surface	flat	flat	sunken

On the basis of phylogenetic tree reconstruction (Fig. 3), species delimitation analyses (Fig. 4) and morphological differentiation (Table 1), we propose that in the *D*. *longan* species complex three species are recognized, one of which is *D*. *longan*, corresponding to clade 2a in Fig. 3. The other two species require nomenclatural changes as follows:

1. *Dimocarpus malesianus* (Leenh.) Lithanatudom & Chaowasku, comb. et stat. nov.
  Basionym: *Dimocarpus longan* ssp. *malesianus* Leenh. In Blumea 19: 1971^5^. This species corresponds to clade 4a of Fig. 3.
2. *Dimocarpus obtusus* ^24^ Lithanatudom & Chaowasku, comb. et stat. nov.

Basionym: Euphoria longana var. obtusa Pierre in Fl. Forest. Cochinch.[Fasc. 20]: 1895 (t. 318)^24^. Homotypic synonym: *Dimocarpus longan* ssp. *longan* var. *obtusus* ^24^ Leenh. In Blumea 19: 1971^5^. This species corresponds to clade 1a of Fig. 3.

DNA barcoding is a tool for species identification^25^ and the result from this study showed the successfully discrimination of the Thao cultivar from other longan samples. In Thailand, the family *Sapindaceae* is divided into 2 species based on various characteristic such as stem, fruit and seed, *etc*. The two species of longan in Thailand consist of the *Euphoria longana* Lamk (synonyms: *Dimocarpus longan* Lour., *Nephelium longana* Cambess) and *Euphoria scandens* Winit Kerr. (synonyms: *Dimocarpus longan* ssp. *longan var*. *obtusus* (Pierre) Leenh)^26, 27^. As the Thao cultivar is defined as *Euphoria scandens* Winit Kerr and is found only in Thailand^3^ the DNA barcoding result from this study supports the proposal that Thao is a longan species different from other longan cultivars in Thailand. The paradoxical classification of longan was described by Choo and Ketsa (1991) who listed two subspecies and five varieties of *Dimocarpus longan*. The classification of Thao cultivar is defined as *Dimocarpus longan* ssp. *longan var*. *obtusus* ^24^ Leenh whereas others longan cultivar in Thailand are the *Dimocarpus longan* ssp. *longan var*. *longan*. From this information the Thao cultivar is classified to be the same subspecies as other longan cultivars but just a different variety^3, 10^. Nevertheless, the DNA barcoding result from our study make the information more clear by supporting that the Thao cultivar should be classified as a different species from other longan cultivars as noted above. This is further supported by Jaroenkit, T.^18^, who noted that the special character of the Thao cultivar was due to its creeping plant-like nature as opposed to others longan cultivar which are perennial plants.

## Methods

### Plant Material and Sampling

**Figure 5:**
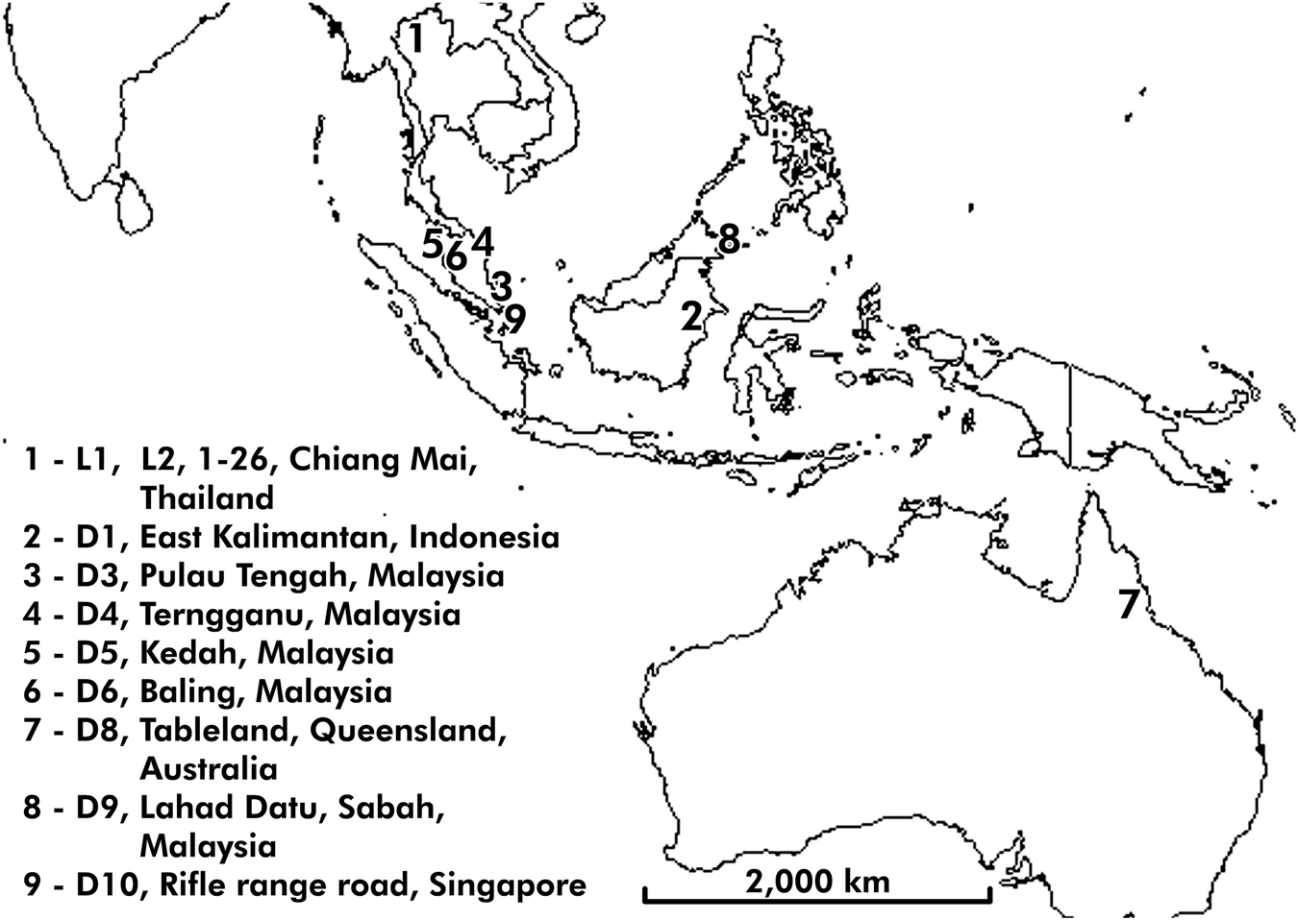
Map of sampling location. Location no. 1 is the location of longan cultivars no. 1–26 and 2 types of lychee (L1 and L2) which have been maintained at Maejo University, Sansai, Chiang Mai, Thailand. Location no. 2–9 represent the location of the herbarium samples. This figure was modified by using the Photoshop program. The source of this figure can be found at https://commons.wikimedia.org/wiki/File:White_World_Map_Blank.png which is licensed under the “Creative Commons Attribution-Share Alike 3.0 Unported” that is free to share (to copy, distribute and transmit the work) and remix (to adapt the work).

A total of 40 samples used in this study consisted of young leaves collected from 26 longan cultivars and 2 types of lychee which have been maintained at Maejo University, Sansai, Chiang Mai, Thailand, and 8 herbarium samples from various locations. The sampling locations are shown in Fig. 5. Sample code, cultivar name and herbarium voucher number of all plant samples are given in Table 2.

**Table 2:** Sample code, cultivar name voucher number and herbarium of plant samples.

Sample number	Name	Cultivar name	Voucher number (Herbarium)
1	*Dimocarpus longan spp*. *longan var*. *longan*	E-Daw	Jaroenkit T, Manochai P, Lithanatudom SK. #1 (CMUB)
2	*Dimocarpus longan spp*. *longan var*. *longan*	Chomphoo	Jaroenkit T, Manochai P, Lithanatudom SK. #2 (CMUB)
3	*Dimocarpus longan spp*. *longan var*. *longan*	BiewKhiew Chiangmai	Jaroenkit T, Manochai P, Lithanatudom SK. #3 (CMUB)
4	*Dimocarpus longan spp*. *longan var*. *longan*	Haew	Jaroenkit T, Manochai P, Lithanatudom SK. #4 (CMUB)
5	*Dimocarpus longan spp*. *longan var*. *longan*	Phuangthong	Jaroenkit T, Manochai P, Lithanatudom SK. #5 (CMUB)
6-1	*Dimocarpus longan spp*. *longan var*. *obtusus*	Thao-1	Jaroenkit T, Manochai P, Lithanatudom SK. #6 (CMUB)
6-2	*Dimocarpus longan spp*. *longan var*. *obtusus*	Thao-2	Jaroenkit T, Manochai P, Lithanatudom SK. #7 (CMUB)
6-3	*Dimocarpus longan spp*. *longan var*. *obtusus*	Thao-3	Jaroenkit T, Manochai P, Lithanatudom SK. #8 (CMUB)
6-4	*Dimocarpus longan spp*. *longan var*. *obtusus*	Thao-4	Jaroenkit T, Manochai P, Lithanatudom SK. #9 (CMUB)
6-5	*Dimocarpus longan spp*. *longan var*. *obtusus*	Thao-5	Jaroenkit T, Manochai P, Lithanatudom SK. #10 (CMUB)
7	*Dimocarpus longan spp*. *longan var*. *longan*	Baidam	Jaroenkit T, Manochai P, Lithanatudom SK. #11 (CMUB)
8	*Dimocarpus longan spp*. *longan var*. *longan*	Phetsakorn	Jaroenkit T, Manochai P, Lithanatudom SK. #12 (CMUB)
9	*Dimocarpus longan spp*. *longan var*. *longan*	Krob-Ka-Ti	Jaroenkit T, Manochai P, Lithanatudom SK. #13 (CMUB)
10	*Dimocarpus longan spp*. *longan var*. *longan*	Jumbo	Jaroenkit T, Manochai P, Lithanatudom SK. #14 (CMUB)
11	*Dimocarpus longan* spp. *longan* var. *longan*	Daw 20	Jaroenkit T, Manochai P, Lithanatudom SK. #15 (CMUB)
12	*Dimocarpus longan* spp. *longan* var. *longan*	Daw 27	Jaroenkit T, Manochai P, Lithanatudom SK. #16 (CMUB)
13	*Dimocarpus longan* spp. *longan* var. *longan*	Daw 75	Jaroenkit T, Manochai P, Lithanatudom SK. #17 (CMUB)
14	*Dimocarpus longan* spp. *longan* var. *longan*	Daw-Kaankhaeng	Jaroenkit T, Manochai P, Lithanatudom SK. #18 (CMUB)
15	*Dimocarpus longan* spp. *longan* var. *longan*	Daw-Luang	Jaroenkit T, Manochai P, Lithanatudom SK. #19 (CMUB)
16	*Dimocarpus longan* spp. *longan* var. *longan*	Daw Kaew Yee	Jaroenkit T, Manochai P, Lithanatudom SK. #20 (CMUB)
17	*Dimocarpus longan* spp. *longan* var. *longan*	Daw-Kaan-Deang	Jaroenkit T, Manochai P, Lithanatudom SK. #21 (CMUB)
18	*Dimocarpus longan* spp. *longan* var. *longan*	Daw-Kaan-On	Jaroenkit T, Manochai P, Lithanatudom SK. #22 (CMUB)
19	*Dimocarpus longan* spp. *longan* var. *longan*	Daw-Lumnam-Ping	Jaroenkit T, Manochai P, Lithanatudom SK. #23 (CMUB)
20	*Dimocarpus longan* spp. *longan* var. *longan*	Daw-Sudhum	Jaroenkit T, Manochai P, Lithanatudom SK. #24 (CMUB)
21	*Dimocarpus longan* spp. *longan* var. *longan*	Daw 13	Jaroenkit T, Manochai P, Lithanatudom SK. #25 (CMUB)
22	*Dimocarpus longan* spp. *longan* var. *longan*	Daengklome	Jaroenkit T, Manochai P, Lithanatudom SK. #26 (CMUB)
23	*Dimocarpus longan* spp. *longan* var. *longan*	Baiyoke	Jaroenkit T, Manochai P, Lithanatudom SK. #27 (CMUB)
24	*Dimocarpus longan* spp. *longan* var. *longan*	Namphueng Tavai	Jaroenkit T, Manochai P, Lithanatudom SK. #28 (CMUB)
25	*Dimocarpus longan* spp. *longan* var. *longan*	Baan-Hong 60	Jaroenkit T, Manochai P, Lithanatudom SK. #29 (CMUB)
26	*Dimocarpus longan* spp. *longan* var. *longan*	Phuen-Mueang	Jaroenkit T, Manochai P, Lithanatudom SK. #30 (CMUB)
D1	*Dimocarpus longan* spp. *malesianus* var. *malesianus*	—	KEP AA 2141 (Herbarium Wanariset East Kalimantan, Indonesia)
D3	*Dimocarpus* sp.	—	KEP 6894 (Flora of pulau Tengah; Malaysia)
D4	*Dimocarpus longan* spp. *malesianus* var. *malesianus*	—	KEP 4343 (Phytochemical Survey of Malaysia Herbarium)
D5	*Dimocarpus fumatus*	—	KEP 4391 (Phytochemical Survey of Malaysia Herbarium)
D6	*Dimocarpus fumatus*	—	KEP 4315 (Phytochemical Survey of Malaysia Herbarium)
D8	*Dimocarpus australianus*	—	KEP 3277 (Herbarium KEP Forest Research Institute Malaysia)
D9	*Dimocarpus longan* spp. *malesianus* var. *echinatus*	—	KEP 116884 (Herbarium of the Forest Department Sandakan)
D10	*Dimocarpus longan* spp. *malesianus* var. *malesianus*	—	SING 2021-231 (Singapore Botanic Gardens Herbarium)
L1	*Litchi chinensis*	Brewster	Jaroenkit T, Manochai P, Lithanatudom SK. #31 (CMUB)
L2	*Litchi chinensis*	Hong Huey	Jaroenkit T, Manochai P, Lithanatudom SK. #32 (CMUB)

### DNA extraction, PCR amplification and sequencing

Total genomic DNA was extracted from young leaves using the cetyltrimethylammonium bromide (CTAB) method^28^. While the herbarium DNA extractions were performed using a CTAB method^29^ modified as according to Bakker^30^. The quantity and quality of the genomic DNA was analyzed by electrophoresis through 1% agarose gels and the 260/280 nm absorbance ratio as determined by spectrophotometry. The genomic DNA was used as a template for PCR amplification using 6 specific primer pairs directed to the nuclear internal transcribed spacer (ITS2)^31, 32^, matK^33^, rbcL^34, 35^, trnH-psbA^36^, trnL (UAA) intron (trnL-i)^37^ and trnL-trn intergenic spacer (trnL-trnF)^38^. Sequences of DNA barcoding primers are shown in Table 3. The PCR amplification step was an initial 95 °C for 5 min followed by 35 cycles of denaturation at 95 °C for 30 sec, annealing for 45 sec and extension at 72 °C for 1 min, and the final extension step was performed at 72 °C for 10 min. The PCR products were run on 1.5% agarose gels in 0.5X TBE buffer. The PCR fragments were visualized under UV light after staining with SYBR SAFE DNA Gel Stain (Invitrogen, U.S.A). The PCR products amplified from the 6 loci were further analyzed by DNA sequencing.

**Table 3:** Primers for PCR amplification.

Primer	Primer sequence (5′ to 3′)	Reference
ITS2	F:ATGCGATACTTGGTGTGAAT	Chen and others^31^
	R:TCCTCCGCTTATTGATATGC	White and others^32^
matK	F:CCCRTYCATCTGGAAATCTTGGTTC	Yu and others^33^
	R:GCTRTRATAATGAGAAAGATTTCTGC	
rbcL	F:ATGTCACCACAAACAGAGACTAAAGC	Levin and others^34^
	R:GTAAAATCAAGTCCACCRCG	Kress and Erickson^35^
trnH-psbA	F:ATTCACAATCCACTGCCTTG	Hajiahmadi and others^36^
	R:ATGGCTTTCAACCTAAATGG	
trnL-i	F:CGAAATCGGTAGACGCTACG	Quemere and others^37^
	R:GGGGATAGAGGGACTTGAAC	
trnL-trnF	F:GGTTCAAGTCCCTCTATCCC	Amundsen^38^
	R:ATTTGAACTGGTGACACGAG	

### Molecular Analyses

The BioEdit Sequence Aligment Editor program^39^ was used to analyze the DNA sequences resulting from sequence analysis of the PCR products generated with the six barcoding primer pairs. The ClustalW program with additional manual curation was used to analyze multiple sequence alignments in order to observe the sequence conservation among longan cultivars.

The nuclear ITS2 region sequence and 5 plastid markers consist of both coding (*matK* and *rbcL* exons) and non-coding regions (*trnH-psbA*, *trnL-trnF* and *trnL-i*) were used to reconstruct the phylogeny. All sequences were checked for ambiguous nucleotide sites and saturation before being subjected to phylogenetic analysis. The uncorrected pairwise distances for transition and transversion substitutions were plotted to visualize saturation and detect the taxa responsible. Analysis of genes separately and in combination was performed using the neighbor-joining (NJ), maximum likelihood (ML)^40^ and Bayesian inference (BI) criteria. jModeltest2.1.1^41^ was used to calculate and determine the best evolutionary substitution model by the Akaike Information Criterion (AIC)^42^ and showed that HKY + G (G = 0.05) model for ITS2, HKY for *matK*, *trnH-psbA*, *trnL-i* and *trnL-trnF*, and JC for *rbcL*.

The incongruence length difference test^43^ in the partition homogeneity test in PAUP 4.0b10 using 100 replicates^22^ were performed to test the concatenated data sets. To assess support at each node, non-parametric bootstrap analyses^44, 45^ were performed using PAUP* version 4.0b10^22^. For coding genes, first and second codon, third codon and all codon positions were tested. The mutation rates were partitioned among genes in concatenated data sets based on model as above. The NJ analysis and the likelihood scores of different data partitions were carried out using PAUP* version 4.0b10^22^ with bootstrap re-sampling^44, 45^ with 1000 replicates. The maximum likelihood^40^ analysis was undertaken using RAxML v. 7.2.7^46, 47^. The bootstrap resampling^44^ with 1000 replicates were performed to support the individual branches of the ML tree.

Bayesian inference (BI) analysis was undertaken using MrBayes version 3.2.5^48^. The 4 chains of a Markov chain Monte Carlo algorithm (MCMC) were used in this criterion. The analysis was run for 10 million generations with a 0.05 heating parameter. The convergence of analysis was estimated using Tracer 1.4.1^49^, and reliable ESS values (>200) were ensured. The sampling was done for every 100 generations and then the first 25% of trees were discarded using a burn-in procedure. Support for nodes was defined as posterior probabilities (P).

The tree topological differences between single-gene phylogenetic trees were compared at the level of resolution obtained by each marker and its bootstrap support. Topological differences of the trees with bootstrap support (BS) and posterior probability (P) less than 75% were not considered. Two lychee cultivars, namely Brewster and Hong Houy were used to root the tree as the out group^50^.

### Bayesian species delimitation

The validity of *Dimocarpus* sp. was re-investigated using three methods of species delimitation analyses: (i) Poisson Tree Processes (PTP)^51^; (ii) Automatic Barcode Gap Discovery (ABGD)^52^; and (iii) Generalized Mixed Yule-Coalescent (GMYC)^40, 53^.

For ABGC^52^, genetic distances between samples were evaluated using the Kimura two parameters (K2P) model, a standard metric in DNA barcoding studies. The ABGD was run via web server http://wwwabi.snv.jussieu.fr/public/abgd/abgdweb.html using default values, except for the relative gap width (X) that was set to 10 to avoid the capture of smaller local gaps.

For PTP and GMYC^53^, all samples of *Dimocarpus* were included. These methods use a phylogenetic input tree from which the fit of speciation and coalescent processes are modeled to delineate a Primary Species Hypotheses (PSHs). The branch lengths were estimated under a relaxed log-normal clock algorithm as an implement in BEAST v1.8.2 package^54^. HKY + G model was applied to construct the tree. The MCMC chains were run for 10 × 10^6^ generations with a sampling step performed for every 100 and 10% burnin. The MCMC output was determined by examination of traces in Tracer 1.6^55^ and analyzed with TreeAnnotator 1.7.4 using all trees after the burnin. A posterior probability limit of 0.5 with maximum clade credibility tree was set. Both the single-threshold and the multiple-threshold versions of the GMYC model^53^ were optimized onto the output tree with the help of the SPLITS v.1.0–19 package for R. The PTP method was executed using the best-scoring ML tree produced earlier using RAxML v. 7.2.7^56^, and was run in Python using the Environment for Tree Exploration package^57^.

### Data availbility statement

The data sets generated and analysed during the current study are available within the paper. All GenBank accession numbers (KY174077-KY174314) o f nucleotide sequences of six loci from individual samples analysed in this study can be retrieved through the NCBI database.

## Conclusion

This is the first report of the genetic relationship of *Dimocarpus* based on multi-locus molecular markers and morphological characteristics. Multiple sequence alignment, phylogenetic tree analysis and species delimitation supported that *Dimocarpus longan* spp. *longan* var. *obtusus* and *Dimocarpus longan* spp. *malesianus* var. *malesianus* should be classified to be different species from *Dimocarpus longan* spp. *longan*. Moreover, sequencing of the DNA barcode revealed the possibility of different species among Thai longan cultivar such as Daw Kaew Yee, Baan-Hong 60 and Phuen-Mueang cultivars. However, more evidence is required to confirm this proposition.
